# The Socio-Economic Cost of Diabetes Mellitus in Korea Using National Health Insurance Claim Data, 2017

**DOI:** 10.3390/healthcare10091601

**Published:** 2022-08-23

**Authors:** Heesun Kim, Eun-Jung Kim

**Affiliations:** 1 National Evidence-Based Healthcare Collaborating Agency, Seoul 04933, Korea; 2 Health, Welfare, Family and Gender Equality Team, National Assembly Research Service, Seoul 07233, Korea

**Keywords:** diabetes mellitus, socio-economic costs, medical expense, indirect cost, non-medical cost

## Abstract

(1) Purpose: As the economy develops and lifestyles become more westernized, diabetes is on the rise in Korea. This study tried to measure the socio-economic cost of diabetes by estimating the direct medical expenses and indirect costs used in Korea during the year due to diabetes mellitus. (2) Methods: This study extracted the insurance claim records from the Korea National Health Insurance claim database to determine the healthcare services provided to patients with diabetes mellitus in 2017. The total diabetes mellitus-related cost was the sum of the direct medical care costs: the costs paid by insurers and patients, the non-covered care costs and the prescribed pharmaceuticals costs, and also the direct non-medical care costs: the transportation costs for visits in outpatients and inpatients and the guardian’s cost for hospitalized patients, as well as the indirect cost: lost productivity. (3) Findings: The total socio-economic cost of diabetic patients in 2017 measured in this study was KRW 3.2 trillion, of which 48.3% was used for medical expenses, 10% was non-medical expenses, and 41.7% was estimated as indirect expenses. (4) Implications: Korea is considered to be aging significantly, and it is considered that more attention should be paid to reducing medical expenses through diabetes management.

## 1. Introduction

Diabetes causes various complications such as cerebrovascular disease, cardiovascular disease, eye disease, and kidney disease. These complications of diabetes show a high prevalence of 30% or more in adult diabetic patients [1]. The population aging in Korea is rapidly progressing to the extent that the elderly population is predicted to reach 35.9% of the population by 2050 [2], with a steadily increasing prevalence of diabetes, and a high complication rate of diabetic patients. Considering this, there is a very high risk that personal and social costs for the treatment and management of diabetic patients will increase sharply in Korea in the not-too-distant future.

Therefore, there is an urgent need to establish policies and improve systems to prevent diabetes by effectively selecting and managing individuals and groups at high risk of developing diabetes in advance, so that the financial burden on the state and households is not rapidly increased due to diabetes.

The prevalence of diabetes in Korea started to show a gradual increase from about 2% in the 1970s, and has reached 10% since the early 1990s. In 2003, 6.4% (approximately 2.19 million people) of adults over the age of 20 had diabetes, and it is expected to increase to 8.3% (3.2 million people) by 2025 [3]. As the national medical expenses for diabetes are also increasing explosively, analyzing and predicting the epidemiologic scale of diabetes has become an unavoidable task in managing the national economy. Since the 1990s, large-scale screening data have been actively used or community cohorts have been established [4,5,6,7]. In-depth research results have been published one after another [8,9,10]. This phenomenon reflects that the keywords of Korean diabetes epidemiologic studies are shifting from simple demographic analysis to in-depth research topics such as environmental, social, and economic factors.

According to the American Diabetes Association (ADA), the direct cost of diabetes in the United States was USD 91.8 billion in 2002, and reached USD 156 billion in 2010 and USD 192 billion in 2020. Furthermore, of the USD 865 billion in healthcare expenditure in the United States, the cost incurred by people with diabetes is USD 160 billion, accounting for 18.5% of the total cost per person. When adjusting for age, sex, and race, it was reported to be about 2.4 times higher [11]. Furthermore, according to a Canadian survey using the same method, direct costs for diabetes care in 1998 amounted to USD 2.6 billion, or 7.8% of Canada’s total healthcare expenditure. A proportion of 50% was spent in hospitals, 19% with outpatients, and 31% on medication [12]. Additionally, in the CODE-2 (Cost of Diabetes in Europe-Type 2 diabetes) study conducted on more than 7000 patients in eight European countries, the group with microvascular complications was 1.7 times higher than the group without complications, and it was reported that the group with complications increased twice as much, and those with all complications increased by 3.5 times [13].

Recent research has been conducted by constructing a cohort in a specific region or through a nationwide data survey. Of course, although there are differences in each research method and subject, it is clear that the prevalence and incidence of diabetes are increasing compared to the past [8,14]. Judging from previous research results, the prevalence of diabetes in Korea in the 2000s is thought to be about 7–10%, and a joint study by the Diabetes Association and the Review and Assessment Service (HIRA) showed a similar rate of 7.7% (based on the age of 20–79). However, if undiagnosed patients are considered, the number of diabetic patients is thought to be higher, and assuming that the prevalence rate by sex–age group in 2003 is maintained, 3.51 million people in 2010 (7.08% of the total population before Statistics Korea estimate), it is expected to rapidly increase to 4.55 million (8.97%) in 2020 and 5.45 million (10.85%) in 2030 [15]. As such, it is expected that socio-economic costs due to diseases, such as medical expenses, will further increase with the increase in the prevalence and rapid aging of the population. Since the healthcare sector, like other sectors, has scarcity of resources, it is necessary to compare inputs and outputs. In the healthcare field, the importance of economic evaluation, which is one of the methods to evaluate both inputs and outputs, is emerging.

This study tried to measure the socio-economic cost of diabetes by estimating the direct medical expenses and indirect costs used in Korea during 2017 due to diabetes. Based on the results of this study, it is expected that the importance of cost reduction through management and control in the pre-diabetic stage of metabolic syndrome can be emphasized by using it as evidence for policy decision making.

## 2. Data and Methods

A characteristic of Korea’s medical insurance system is that it is a national health insurance system that takes the form of social insurance and is operated by a single insurer for the entire nation. Unlike the national health services provided by other countries, Korea’s medical insurance system is financed by insurers. Accordingly, the characteristics that appear include the obligation to purchase insurance and pay premiums, charge premiums according to the ability to pay, and equal coverage.

Health insurance in Korea is closely related to the medical system. This is because health insurance determines the items for medical expenses between the male health insurance member (patient) and the health insurance beneficiary (doctor and medical institution). In general, the patient receives a portion of the medical expenses for items covered by health insurance, and the rest is received by requesting the Health Insurance Corporation. At this time, the payment is made after being reviewed by the Health Insurance Review and Assessment Service under the Ministry of Health and Welfare.

In addition, since the national health insurance subscription rate is 97%, and the majority of people are enrolled, this study using the national health insurance claim data is highly accurate in measuring the national healthcare costs.

In this study, we estimated the economic cost of DM using a prevalence-based approach. For the year 2017 (1 January 2017–31 December 2017), we did not separate the incidence cohort from prevalence cohort and calculated medical cost due to DM in Korea. The claim data were extracted from the server of the National Health Insurance Corporation in a state of randomized personal identification data and downloaded to the author’s PC for analysis. A cohort of patients affected by prevalence of diabetes mellitus cost-of-illness approach was adopted in order to estimate the utilization of resources and calculate costs from a societal perspective: direct medical care cost, direct non-medical care cost (formal or informal care), and productivity losses (i.e., indirect costs) were measured. In order to measure medical expenses based on the prevalence, we included all patients who were hospitalized or outpatients with diabetes in 2017. However, in the case of outpatient visits, only patients who visited 3 or more times with the same ICD-code were included as subjects of analysis to exclude suspected patients. 

The ICD-10 (International Classification of Disease 10th version) code of DM is ‘E10–14′ so we requested insurance claim data about ‘E10–14′ of the National Health Insurance Corporation including prevalence cases, and the cost of using medical services. The subject of this study was patients diagnosed with DM as a main disease in 2017. To calculate socio-economic cost, we classified direct costs and indirect costs [16]. A direct cost, which was paid for curing diseases, included direct medical care costs and direct non-medical care costs. Direct medical care costs included costs paid by insurers, paid by patients, prescribed pharmacy cost, and so on. Direct non-medical care costs were constituted by transportation cost for visiting hospitals and guardian’s cost. An indirect cost meant lost productivity as an opportunity cost of time visiting hospital or premature death.

Table 1 lists the detail cost items and the detailed variables. We classified the socio-economic costs of DM using Han et al.’s comprehensive approach [17]. The total costs were composed of direct and indirect healthcare costs and indirect costs. The direct healthcare costs consisted of inpatient costs, outpatient costs, and pharmaceutical costs. For the pharmaceutical costs, due to the nature of the data, it is difficult to confirm the list of all drugs used, and considering the drug prescription characteristics of patients suffering from various complex diseases other than diabetes, it is difficult to extract only the exact drug costs related to diabetes, so drug costs were excluded. Therefore, we extracted the prescription claim database for DM patients for 2017 from the drug claims from the Health Insurance Review and Assessment Service. In Korea, the pharmaceutical cost of inpatients was included in admission cost. Therefore, we considered pharmaceutical cost separately at the outpatient section only.

The direct non-medical care cost consisted of the transportation cost and caregiver’s costs [17,18]. The monthly admission rate and outpatient clinic visiting rate were obtained from the number of admissions and outpatient visits divided by the total patients in each month. In the transportation cost, it was assumed that one caregiver accompanied a patient when the patient was admitted or visited the outpatient clinics. This was calculated using data from the Korean Health Panel Study, which was surveyed by the government in 2017 [19]. In Korea, there is a caregiver system that takes care of inpatients and stays with inpatients instead of families. Since market prices are established for these services, the cost of caregivers is added only for inpatients.

Indirect costs are defined as “output lost because of cessation or reduction of productivity due to morbidity or mortality [20].” In this study, indirect costs consisted of premature death costs and patients’ opportunity cost based on the human capital approach of opportunity cost using market payment [21,22,23]. The cost due to productivity loss was defined as the patients’ productivity loss associated with the outpatient visits or hospitalization [24]. The premature death cost was defined as patients who died prematurely. Therefore, how much money they would have earned if they had lived until the average retirement age was calculated. For discount, we accept 3% as a rate to reflect the present value of future earnings [25].

This study was conducted using public data from NHI and HIRA and did not have personal identifiers. This data publishing was approved by each organization’s ethics committee (KU-IRB-18-EX-51-A-1). The data were extracted, collected, calculated, and analyzed using Microsoft Excel and SAS 9.2 (SAS Institute, Inc., Cary, North Carolina, USA).

## 3. Results

The characteristics of the diabetes mellitus cohorts are shown in Table 2. In this study, we had 2,153,594 (51.8%) male and 2,003,924 (48.2%) female patients; old-old (age ≥ 60 years) accounted for 49.7% of all. Almost all of the diabetes mellitus patients had type 2 diabetes (89.5%). The proportion of diabetes patients who had been hospitalized was 23.98%, and outpatient visits accounted for 95.68% (Table 2).

In the case of direct medical expenses, the overall cost of outpatients was more than three times higher than that of inpatients. Due to the nature of the disease, it is considered that the cost of the outpatient visit is rather high because it is chronically managed through outpatient visits and requires medication. Most of the hospitalized patients were type 2 diabetes patients. This is thought to be because there are many elderly patients who failed to control their blood sugar because they did not properly improve their lifestyle. 

In the case of women, outpatient medical expenses accounted for 44.37% of the socio-economic cost, and took up the largest share. There are studies that see this difference as a high BMI in women [26,27,28]. Due to this, women have a higher risk of CVD than men and are more likely to take antihypertensive drugs, making it more difficult to maintain normal HbA1c and LDL levels than men. In addition, risk factors that are prominent in women, such as sex hormone imbalance or lower energy metabolism rate, also have an effect. In the case of direct medical expenses, the proportion of transportation expenses among women and men was the lowest at 1–1.3%, and in the case of caregivers, women spent KRW 15 million more than men. In the case of indirect costs, productivity loss due to premature death accounted for 38.18% of men’s socio-economic cost, accounting for the largest portion, whereas for women, it was 19.79%, which was about KRW 450 billion lower than men. This is thought to be because the average wage of men is higher than that of women. The total socio-economic cost of diabetic patients in 2017 measured in this study was KRW 3.2 trillion, of which 48.3% was used for medical expenses, 10% was non-medical expenses, and 41.7% was estimated as indirect expenses (Table 3).

## 4. Discussion

This study aimed to measure the current total economic cost of DM in Korea to inform decision makers and payers. The total economic cost in 2017 turned out to be significant and the total cost was estimated at KRW 3.2 trillion. Of these total costs, 48.3% were direct medical expenses and 41.7% were attributable to indirect costs (loss of productivity due to hospital visits and loss of productivity due to premature death). Therefore, strategies to reduce these indirect costs can result in significant cost savings. Moreover, an aging population and changing lifestyles are likely to further increase the total economic cost of diabetes [29,30].

The elderly population in Korea is increasing at a much faster rate than other countries. Although discussions about aging policies continue to emerge, it has been pointed out that there is a limit to being dealt with from a welfare point of view. In 2018, the elderly population accounted for 14.3% of the total population in Korea, entering a solid society. Looking at the trend, it is expected that by 2025, we will enter a super-aged society where the proportion of the elderly population will be 20%, and the crisis of a population cliff will come quickly. The older they are, the more they have chronic diseases, the more they need healthcare, and their ability to perform daily life is different. As you age, your healthcare costs increase, and so does the poverty rate. As society as a whole is aging, not just the poor, it is necessary to create an environment in which the elderly can act as subjects. In order to respond to the aging society, health policy should establish long-term and step-by-step plans from a macro-perspective in the tunnel of aged society. First of all, it is necessary to recognize the elderly as a target of health policy as an economic entity that requires a significant amount of medical care, not as a minority of vulnerable groups of welfare targets.

The results of this study can be summarized in a few key points as follows.

First, comparing with the results of other studies conducted in Korea, direct medical expenses for type 2 diabetes account for 10.6% of total medical expenses (calculated only as direct medical expenses excluding out-of-pocket expenses [31]). This is higher than the rate of type 2 diabetes in France (5%) [32], and the economic burden of diabetes is becoming a major public health problem in Korea. On the other hand, diabetic patients are 1.7 times more likely to have vascular complications, 3.1 times more likely to suffer from heart disease, 15 to 40 times more likely to have a foot amputation, and have a higher premature death rate than those without diabetes [33]. Among these complications, macrovascular complications such as heart disease and stroke are known to be the main causes of death in diabetic patients [13,34], and diabetic patients are three times more likely to be hospitalized and five times more expensive than those without diabetes [33]. Therefore, if the socio-economic cost is measured in consideration of the complications, the value will be greatly increased compared to the current result.

Second, the outpatient cost accounted for 37.7% of the total medical expenses of diabetic patients. In the US study, the hospitalization cost was the highest for diabetic patients compared to non-diabetic patients because the risk of hospitalization was high and hospitalization was frequent [34]. It was different to this study. However, in the Italian study [13], when considering both the presence and number of complications of diabetes, the total drug swallow accounted for the largest proportion, from 46% to 66%. This can be seen as a difference in the composition of medical expenses due to differences in insurance systems in each country and differences in access to hospitals. In this study, about 10% of socio-economic costs are used for hospitalization, and if indirect costs related to productivity loss due to hospitalization or death are considered, medical expenses due to hospitalization are considered to drive socio-economic costs of diabetes. Therefore, it is recommended to set the direction of treatment in three aspects: (1) continuity of prescription, (2) number of outpatient visits, and (3) continuity of outpatient visits, while gradually reducing inpatient treatment for diabetic patients [35].

In this study, we have some limitations. The possibility of underestimation should be considered because the number of diseased-but-undiagnosed patients cannot be confirmed. However, since Korean medical institutions are required to subscribe to the health insurance system by designation system, if a patient visits a medical institution, you can be sure that all of them are included in these data, and it is believed that there is no possibility that the private sector will incur medical expenses on its own. Furthermore, since the data used in this study were extracted based on the main diagnosed disease when claiming the data, diabetic patients were judged to be present when they visited the outpatient clinic three or more times in a year, and they were judged to be present when they were hospitalized more than once. Due to the nature of the disease and diabetes being managed on an outpatient basis, there were more cases of hospitalization than outpatients with age, regardless of whether it was type 1 diabetes or type 2 diabetes. This phenomenon is thought to be due to the comorbidity or multi-morbidity that people have with diabetes as they age. However, analyzing the characteristics of inpatient and outpatient ICD-10 codes is for a future study.

Considering the rate of aging in Korea, the cost for the treatment of chronic diseases including diabetes occupies a large proportion in terms of national medical expenses. Furthermore, considering the huge socio-economic medical expenses involved, this study can be used as a basis to emphasize the need for a preventive policy for chronic diseases. It is hoped that through the results of this study, the direction of social insurance can be reset by asserting the need for primary care-centered chronic disease prevention. The necessity of introducing a social program for the management of chronic diseases such as diabetes is emphasized as continuous outpatient use will reduce hospitalization and death and reduce medical costs.

## Figures and Tables

**Table 1:** Detailed variables and data sources per cost item.

Cost	Items	Detailed Variables
Direct health cost (DHC)	Inpatient costs	Average inpatient healthcare costs (IC) Non-Benefit rate—inpatient (NBRi)
	Outpatient costs	Average outpatient healthcare costs (OC) Non-Benefit rate—outpatient (NBRo) Outpatient Pharmaceutical costs (OPC)
Direct non-medical care costs (DNC)	Transportation fee	Average admission rate (AR) One-way transportation fee for an inpatient (Ti) Average outpatient visit rate (OR) One-way transportation fee for an outpatient (To)
	Caregiver costs	Average days of admission (D) Average number of outpatient visits (NO) Average wage in a day (Wd)
Indirect cost (IDC)	Productivity loss	Average days of admission (DP) Average number of outpatient visits (NOP) Average wage in a day (WPd) Average percentage of employment (Ey)
	Premature death cost	Average wage in a year per age group (Wy) Average retirement age (Ra) Number of deaths per age group (Da) Interest rate (r)

**Table 2:** Characteristics of patient cohort.

Characteristics		N (Mean)	% (sd)
Total number of diabetes mellitus patients		4,157,518	100
Gender	Male	2,153,594	51.8
	Female	2,003,924	48.2
Age	<60	2,091,232	50.3
	≥60	2,066,286	49.7
Type of Diabetes Mellitus ^1^	Type 1 (E10)	128,883	3.1
	Type 2 (E11)	3,720,979	89.5
	Others (E12–E14)	307,656	7.4
Healthcare utilization	Inpatient cohort ^2^	997,157	23.98
	Outpatient cohort ^2^	3,978,115	95.68
	Inpatient days per patient, mean (SD)	8	51.22

^1^ Divided by main diagnosed ICD-10 code ^2^ Duplicated count case.

**Table 3:** Total estimated costs according to gender of DM (unit: KRW 1000).

Cost Components		Costs					
		Female		Male		Total	
		Costs	Percent (%)	Cost	Percent (%)	Cost	Percent (%)
Direct health cost	Inpatient costs	174,209,574	12.44%	175,354,015	9.28%	349,563,589	10.60%
	Outpatient costs	621,452,688	44.37%	617,951,679	32.71%	1,239,404,367	37.70%
Direct non-medical care costs (DNC)	Transportation fee	18,866,214	1.35%	18,846,141	1.00%	37,712,355	1.10%
	Caregiver costs	153,622,279	10.97%	138,513,226	7.33%	292,135,505	8.90%
Indirect cost (IDC)	Productivity loss	155,261,173	11.09%	217,246,713	11.50%	372,507,886	11.30%
	Premature death cost	277,101,768	19.79%	721,422,453	38.18%	998,524,221	30.40%
Total		1,400,513,696	100.00%	1,889,334,227	100.00%	3,289,847,923	100.00%

## Data Availability

The dataset analyzed during the current study is not publicly available due to Korean information security law.
